# Identification and expression analysis of *GARP* superfamily genes in response to nitrogen and phosphorus stress in *Spirodela polyrhiza*

**DOI:** 10.1186/s12870-022-03696-5

**Published:** 2022-06-25

**Authors:** Xuyao Zhao, Jingjing Yang, Xiaozhe Li, Gaojie Li, Zuoliang Sun, Yan Chen, Yimeng Chen, Manli Xia, Yixian Li, Lunguang Yao, Hongwei Hou

**Affiliations:** 1 The State Key Laboratory of Freshwater Ecology and Biotechnology, The Key Laboratory of Aquatic Biodiversity and Conservation of Chinese Academy of Sciences, Institute of Hydrobiology, Chinese Academy of Sciences, Wuhan, 430072 China; 2 University of Chinese Academy of Sciences, Beijing, 100049 China; 3 Henan Key Laboratory of Ecological Security for Water Source Region of Mid-Line of South-to-North Diversion Project of Henan Province, Collaborative Innovation Center of Water Security for Water Source Region of Mid-Line of South-to-North Diversion Project of Henan Province, Nanyang Normal University, Nanyang, 473061 China

**Keywords:** *Spirodela polyrhiza*, *GARP* superfamily, NIGT1/HRS1/HHO transcription factor, PHR transcription factor, Expression pattern

## Abstract

**Background:**

GARP transcription factors perform critical roles in plant development and response to environmental stimulus, especially in the phosphorus (P) and nitrogen (N) sensing and uptake. *Spirodela polyrhiza* (giant duckweed) is widely used for phytoremediation and biomass production due to its rapid growth and efficient N and P removal capacities. However, there has not yet been a comprehensive analysis of the *GRAP *gene family in *S. polyrhiza*.

**Results:**

We conducted a comprehensive study of *GRAP* superfamily genes in *S. polyrhiza*. First, we investigated 35 *SpGARP* genes which have been classified into three groups based on their gene structures, conserved motifs, and phylogenetic relationship. Then, we identified the duplication events, performed the synteny analysis, and calculated the *K*_*a*_/*K*_*s*_ ratio in these *SpGARP* genes. The regulatory and co-expression networks of SpGARPs were further constructed using *cis*-acting element analysis and weighted correlation network analysis (WGCNA). Finally, the expression pattern of *SpGARP* genes were analyzed using RNA-seq data and qRT-PCR, and several NIGT1 transcription factors were found to be involved in both N and P starvation responses.

**Conclusions:**

The study provides insight into the evolution and function of *GARP* superfamily in *S. polyrhiza,* and lays the foundation for the further functional verification of *SpGARP* genes.

**Supplementary Information:**

The online version contains supplementary material available at 10.1186/s12870-022-03696-5.

## Background

GARP (G: GOLDEN2 in *Zea mays*, AR: ARR-B in *Arabidopsis thaliana*, P: Psr1 in *Chlamydomonas reinhardtii*) is a plant specific transcription factor (TF) superfamily with diverse functions involved in nutrients (N and P) sensing, chloroplast development, circadian clock oscillation, and hormonal signaling [1, 2]. GARP superfamily TFs are made of type-B authentic response regulators (ARR-B) and GOLDEN2-like (G2-like, GLK) TFs, and first be named by Riechmann in *A. thaliana* [1, 2]. Both of these two TFs contain GARP motif (also named B-motif), which is a domain of about 60 amino acids that forms a three α-helices 3D structure containing a helix-turn-helix (HTH) motif [2, 3]. B-motif is the multifunctional domain in GARP proteins that responsible for both nuclear localization and DNA binding [2, 3].

*GOLDEN2* gene was first reported to function in chloroplast biogenesis in *Z. mays *[4]. Then, the orthologues of *GOLDEN2* were identified in *A. thaliana*, *Physcomitrella patens*, *Capsicum annuum*, *Oryza sativa*, and *Solanum lycopersicum* [4–8]. *AtGLK1/2* in *A. thaliana* are the orthologous genes of *ZmGOLDEN2*, expressing in photosynthetic tissue and exhibit functional redundancy, however, the double mutants reduce the accumulation of photosynthetic gene products and thylakoids in chloroplasts [7]. *ZmGOLDEN2* and their orthologues in *O. sativa* and *A. thaliana* are regulated by light [6, 7]. Subsequently, GOLDEN2 and its orthologues were classified into the GARP family with ARR-B and Psr1-like genes [1]. A gene can be classified in the GARP family if the derived protein contain B-motif [2]. ARR-B proteins contain a B-motif in their C-terminal, act as positive regulators in the two-component cytokinin signaling pathway and play pivotal roles in plant development, including vascular development, light sensitivity, chlorophyll production, hypocotyl elongation, and cell division in the root and shoot [9]. GLK TFs play important roles in biotic and abiotic stresses, such as pathogen infection, salt stress, drought, and nutrient deficiency. AtGLK1 regulates genes that involved in disease resistance and effect on different pathogen [10], nine *AtGLK* genes were responded to salt stress in *A. thaliana* [11]. Both of Phosphorus Starvation Response 1 (Psr1) in *C. reinhardtii* and Phosphate Starvation Response 1 (PHR1) in *A. thaliana* play the central regulatory roles in inorganic phosphate (Pi) sensing system [12, 13].

The duckweed family (*Lemnaceae*) is a group of fast-growing free-floating aquatic plants, and distributed in various fresh water environments throughout the world besides the most extreme habitats [14]. Based on the morphological characteristics and molecular taxonomy, 36 duckweed species were recognized belonging to five genera: *Spirodela* (2), *Landoltia* (1), *Lemna* (12), *Wolffiella* (10), and *Wolffia* (11) [15, 16]. Duckweed is an attractive model in plant research for the convenient cultivation system, clear genetic background, and robust transformation methods [17]. They are some of the fastest growing flowering plants (doubling time < 30 h under the optimal growth conditions) in the world, with high productivity of dry mass (80–100 tons per hectare per year) which is more than five times that of maize [18–20]. Duckweed also exhibits efficient N and P removal capacities from wastewater with about 1.3 g•m^−2^•d^−1^ and 0.18 g•m^−2^•d^−1^ respectively [21]. Thus, duckweed is also considered as ideal plant in phytoremediation to recover nutrients (N and P) from eutrophic water. *S. polyrhiza* occupies the ancestral phylogenetic position among duckweeds, possesses the largest individual and the smallest genome size in the *Lemnaceae* [22]. The prominent performance of *S. polyrhiza* in nutrient removal from wastewater has been observed in previous studies [23, 24]. The available genomic data and robust transformation method of *S. polyrhiza* provide theoretical and technical supports for the research of molecular mechanism and germplasm improvement in *S. polyrhiza* [25–30]. Thus, *S. polyrhiza* is one of the best prospective species in *Lemnaceae* for phytoremediation and biomass production.

N and P are two major essential elements required for plant growth. Nitrate (NO_3_^−^) and ammonium (NH_4_^+^) are the main forms of N source in soils, and inorganic phosphate (Pi) is the main form of P source for plant uptake in the environment [31, 32]. The distributions of N and P in the environment are affected by many factors which create a variable spatiotemporal landscape at the local and global scale [33]. Thus, it is crucial for plants to adapt themself to various nutrient environments (N and P content), however, the underlying molecular mechanism remains to be elucidated in-depth. The GARPs TFs are involved in the responses to nutrients and include probable nutrient sensors of plants. CrPsr1 is the first reported GARP TF involving in nutritional responses, it is critical for the acclimation of *C. reinhardtii* to P starvation [12]. AtPHR1 was the central regulator in the downstream of Pi starvation signaling pathway [13, 34]. Then, Nitrate-Inducible Garp-Type Transcriptional Repressor 1 (NIGT1)/Hypersensitivity to Low Phosphate-Elicited Primary Root Shortening 1 (HRS1)/HRS1 Homolog (HHO) were found to be the most robustly and quickly NO_3_^−^ regulated genes [35–37]. Furthermore, NIGT1 TFs are involved in both N and P sensing, uptake, and assimilation through the interactions with PHR1, Nodule Inception (NIN)-like protein (NLP), SYG1-Pho81-XPR1 (SPX) domain proteins, phosphate transporter 1 (PHT1), nitrate transporter 1 (NTR1), and NRT2 [38, 39].

The *GARP* superfamily or *GLK* subfamily has been identified and characterized in *A. thaliana*, *O. sativa*, *Z. mays*, *Nicotiana tabacum*, and *Gossypium hirsutu*m [1, 40–43]. Bhutia et al. (2020) found that some GARP members (OsGLK10, OsGLK15, OsGLK22, and OsGLK30) respond to P starvation in rice [41]. However, the *GARP* gene family has not been thoroughly examined in *S. polyrhiza*, to the best of our knowledge. In current study, we perform a comprehensive analysis of GARP superfamily in the giant duckweed *S. polyrhiza*, including chromosomal locations, evolutionary perspectives, structural arrangement, and their functional role through gene expression analysis. The results of this study offer a robust platform for further functional studies of the candidate *SpGARP* genes, so as to understand the molecular mechanism of N/P sensing, acquisition and balance, and enable their efficient use in the germplasm improvement to enhance the phytoremediation potential of *S. polyrhiza*.

## Results

### Genome‑wide identification of *GARP* superfamily genes in *S. polyrhiza*

The GARP proteins in Arabidopsis and rice were used as queries to perform the BLASTP search in *S. polyrhiza* 7498 genome databases. To mine all the potential GARP proteins that harbored B-motif in *S. polyrhiza*, the Hidden Markov Model (HMM) profile of B-motif was built using HMMER 3.3.2 based on the identified SpGARPs [44], and used for further search of SpGARP proteins. A total of 35 *GARP* genes were identified in *S. polyrhiza*, including 7 *SpARR-B* and 28 *SpGLK* genes. The detailed information of *SpGARP* genes was listed in Table 1, including gene ID, genomic position, gene length, exon number, protein length, molecular weight (MW), isoelectric point (pI), instability index, grand average of hydropathicity (GRAVY) and subcellular localization. The *GARP* genes were named as *SpARR-B1-7* and *SpGLK1-28* according to their chromosomal locations. The length of SpGARP proteins ranged from 229 (SpGLK17) to 693 amino acids (aa) (SpARR-B2), with the average of 386 aa, and their MW ranged from 25.47 (SpGLK17) to 74.33 kDa (SpARR-B3). The pIs were around 5.0 in SpARR-B family, and ranged from 5.2 to 9.6 in GLK family. The instability index ranged from 39.61 (SpGLK21) to 75.66 (SpGLK13). The GRAVY ranged from -0.327 (SpARR-B2) to -0.964 (SpGLK1). SpARR-B6 was predicted to be localized at the extracellular space for the 14 aa signal peptides (SP) (MAASILSLFPGGLG) in the N-terminal while other 34 SpGARP proteins were localized at the nucleus. To examine the evolutionary relationships of *SpGARP* genes, GARPs in two close related species *Colocasia esculenta* (10 *CeARR-B* and 35 *CeGLK* genes) and *Wolffia australiana* (5 *WaARR-B* and 23 *WaGLK* genes) were identified (Table S1).

**Table 1:** Characteristic features of SpGARPs

S. No	Gene ID	Protein length (aa)	Exons	Molecular weight (kDa)	Theoretical pI	Instability index	Grand average of hydropathicity	Subcellular location
SpARR-B1	Spo004408	693	5	73.98	6.24	53.21	-0.327	nucleus
SpARR-B2	Spo001985	541	5	60.87	5.66	44.75	-0.523	nucleus
SpARR-B3	Spo006367	689	6	74.33	5.41	46.65	-0.439	nucleus
SpARR-B4	Spo007463	468	6	52.79	6.06	51.10	-0.537	nucleus
SpARR-B5	Spo008950	575	6	63.58	4.63	58.94	-0.504	extracellular space
SpARR-B6	Spo008951	527	6	58.10	5.18	56.33	-0.518	nucleus
SpARR-B7	Spo012555	526	11	57.50	5.85	64.41	-0.588	nucleus
SpGLK1	Spo000053	352	5	39.32	9.53	59.96	-0.964	nucleus
SpGLK2	Spo000614	356	1	37.80	6.57	64.92	-0.517	nucleus
SpGLK3	Spo003067	459	7	49.72	5.20	70.60	-0.628	nucleus
SpGLK4	Spo003413	294	6	32.47	6.67	49.39	-0.714	nucleus
SpGLK5	Spo005377	297	6	32.66	7.01	47.10	-0.665	nucleus
SpGLK6	Spo005389	376	5	40.46	6.49	73.16	-0.779	nucleus
SpGLK7	Spo001652	367	4	40.24	9.55	54.31	-0.402	nucleus
SpGLK8	Spo008782	322	1	34.54	6.43	54.53	-0.437	nucleus
SpGLK9	Spo010181	242	4	26.12	9.32	65.94	-0.617	nucleus
SpGLK10	Spo007174	433	6	46.84	5.66	60.43	-0.718	nucleus
SpGLK11	Spo007312	503	6	55.74	7.05	59.69	-0.808	nucleus
SpGLK12	Spo010995	411	7	44.39	6.13	57.49	-0.721	nucleus
SpGLK13	Spo011612	351	4	38.67	7.09	75.66	-0.649	nucleus
SpGLK14	Spo011622	282	6	30.84	8.98	59.36	-0.678	nucleus
SpGLK15	Spo008930	283	6	31.65	7.80	60.46	-0.622	nucleus
SpGLK16	Spo009589	239	6	25.73	9.59	75.36	-0.753	nucleus
SpGLK17	Spo012149	229	6	25.47	9.27	60.33	-0.669	nucleus
SpGLK18	Spo012150	246	6	27.39	8.48	45.72	-0.574	nucleus
SpGLK19	Spo012405	277	6	30.73	9.07	41.16	-0.565	nucleus
SpGLK20	Spo012640	306	4	33.94	8.53	61.63	-0.730	nucleus
SpGLK21	Spo012709	315	4	34.12	9.60	39.61	-0.555	nucleus
SpGLK22	Spo012971	307	6	32.82	8.68	55.89	-0.581	nucleus
SpGLK23	Spo013101	310	6	34.83	9.32	67.00	-0.904	nucleus
SpGLK24	Spo013406	481	8	52.79	5.63	68.13	-0.740	nucleus
SpGLK25	Spo014991	428	5	46.96	6.45	73.48	-0.840	nucleus
SpGLK26	Spo017076	269	6	29.81	8.83	56.22	-0.599	nucleus
SpGLK27	Spo018029	318	5	35.72	5.92	62.48	-0.815	nucleus
SpGLK28	Spo018160	432	4	47.01	5.71	55.43	-0.716	nucleus

### Phylogenetic analysis of GARP superfamily genes in five species

**Fig. 1:**
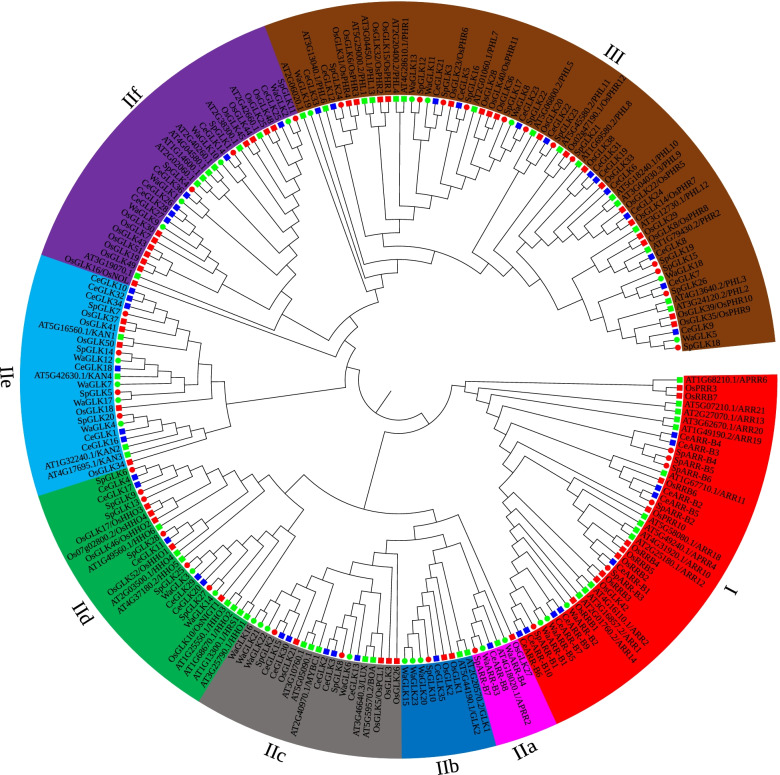
Phylogenetic trees and subgroup classification of GARP proteins in *A. thaliana*, *C. esculenta*, *O. sativa*, *S. polyrhiza*, and *W. australiana*. The phylogenetic tree was constructed by the neighbor-joining (NJ) method with 1000 bootstrap replicates. The green rectangle, blue rectangle, red rectangle, red cycle and green cycle represent GARP proteins from* A. thaliana*, *C. esculenta*, *O. sativa*, *S. polyrhiza*, and. *W. australiana*

The full-length amino acid sequences of *SpGARPs*, *AtGARPs* (14 *AtARR-B* and 41 *AtGLK* genes), *OsGARPs* (10 *OsARR-B* and 47 *OsGLK* genes), *CeGARPs* and *WaGARPs* were subjected to multiple sequence alignments using ClustalW [45]. The unrooted neighbor-joining (NJ) phylogenetic tree was divided into eight branches belonging to three big groups (I, IIa-f and III). As shown in Fig. 1, the majority of ARR-B proteins were clustered into the group I, besides that, six members (At4G12020.1, CeARR-B8, OsGLK27, SpARR-B7, WaARR-B3, and WaARR-B4) were clustered into clade IIa. The orthologous genes of *ZmGOLDEN2* in five species were clustered into the subgroup IIb. Both of Arabidopsis and rice possess two members, *C. esculenta* and *S. polyrhiza* have a single copy in their genome, and three orthologous genes were present in the smallest and simplest flowering plant *W. australiana*. The findings indicate the unusual evolutionary pathways of ZmGOLDEN2 orthologous genes in Araceae plants, especially in the duckweed plants. A total of 20 *GLK* genes (5 *AtGLKs*, 5 *CeGLKs*, 4 *OsGLKs*, 2 *SpGLKs* and 4 *WaGLKs*) were clustered into subgroup IIc. The members in subgroup IIc were involved in circadian oscillation, including *AtLUX* (*AT3G46640.3*), *AtBOA* (*AT5G59570.2*), *AtMYBC1* (*AT2G40970.1*), *OsPCL* (*OsGLK5*), *OsPCL-like* (*OsGLK21*) genes in Arabidopsis and rice [41]. The members of 25 NIGT1/HRS1/HHO (also named NIGT1) TFs were clustered into subgroup IId. It has been found that NIGT1 TFs coordinated N and P responses in Arabidopsis [38, 39, 46–48]. Six *SpGLK* genes belonged to NIGT1 subfamily and were named based on their topological locations in the phylogenetic tree: *SpHHO1* (*SpGLK6*), *SpHHO2* (*SpGLK9*), *SpHHO3* (*SpGLK13*), *SpHHO4*/*SpNIGT1.1* (*SpGLK25*), *SpHHO5*/*SpNIGT1.2* (*SpGLK27*), and *SpHHO6* (*SpGLK28*). Subgroup IIe harbored 23 *KANADI* (*KAN*) genes which play key roles in organ positioning, cell type patterning and organ morphogenesis of shoot apical meristem (SAM) [49, 50]. Subgroup IIf contained 35 members which functioned to leaf development [41], including 7 AtGLKs, 11 OsGLKs, 6 CeGLKs, 3 SpGLKs and 4 WaGLKs. All the Phosphate Starvation Response (PHR)/PHR1-like (PHL) TFs were clustered into group III, *SpGLK3* (*SpPHR1*) and *SpGLK12* (*SpPHR2*) exhibited closer relationships to *AtPHR1* and *OsPHR2*, which were the center regulator of phosphate starvation response (PSR) in Arabidopsis and rice, respectively [13, 51, 52].

### Chromosomal locations, duplication analysis, and synteny analysis of *SpGARP* genes

**Fig. 2:**
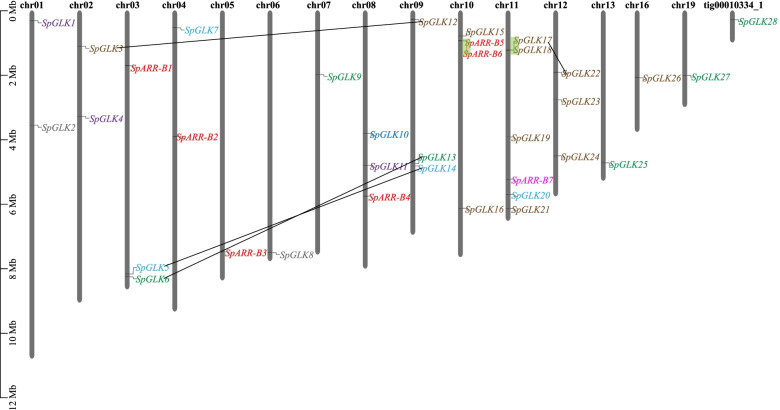
Chromosomal distribution of *SpGARP* genes. Chromosomal mapping was based on the physical position in 15 chromosomes and one contig. Genes belonging to the same group are indicated by the same color. Segmental duplications are marked with black solid line, tandem duplications are marked with green background

As shown in Fig. 2, a total of 35 *SpGARP* genes distributed in 15 chromosomes and one contig (tig00010334_1, *SpGLK28*), most chromosomes contain 1–3 *GARP* genes except chr10 (4 *SpGARP* genes, *SpGLK15/16* and *SpARR-B5/6*) and chr11 (6 *SpGARP* genes, *SpGLK17-21* and *SpARR-B7*). The duplication events of *SpGARP* genes were analyzed using McScanX [53]. Four segmental duplication events (*SpGLK3*-*SpGLK12*, *SpGLK5*-*SpGLK14*, *SpGLK6*-*SpGLK13* and *SpGLK17*-*SpGLK22*) and two tandem duplication events (*SpARR-B5*-*SpARR-B6*, *SpGLK17*-*SpGLK18*) were identified, all the duplicated gene pairs belong to the same group.

**Fig. 3:**
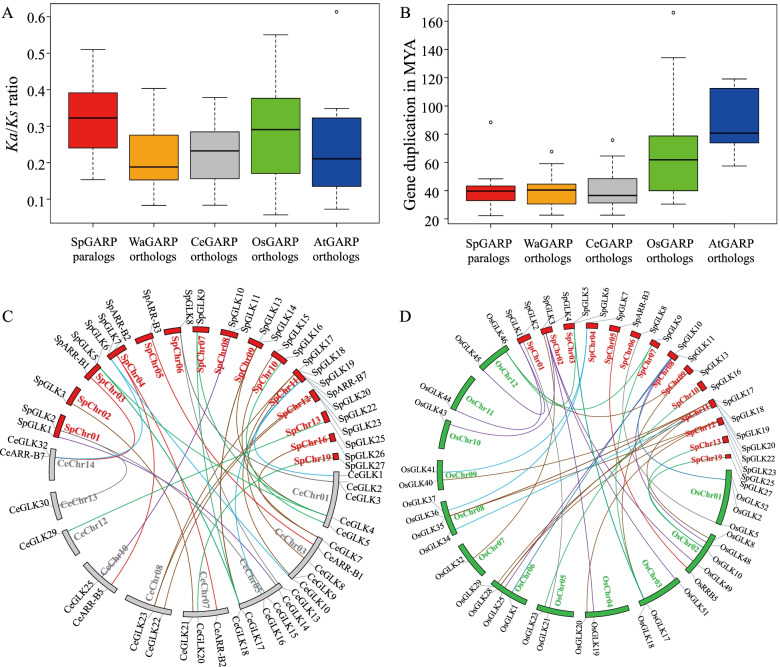
Inference of *SpGARP* gene duplications. **A** Box and whisker plots representing the distribution of *K*_*a*_*/K*_*s*_ ratios in *SpGARP* paralogs and orthologs. **B** Estimated gene duplication time of *SpGARP* paralogs and orthologs in MYA. **C** Syntenic relationship of *SpGARP* orthologs in chromosomes of *C. esculenta*. **D** Syntenic relationship of *SpGARP* orthologs in chromosomes of *O. sativa*

A total of 10 *SpGARP* paralogs gene pairs were identified based on the stringent amino acid sequence homology analysis (Table S2). We also identified the orthologous relationship of *SpGARP* genes with *A. thaliana* (8 gene pairs), *C. esculenta* (42 gene pairs), *O. sativa* (49 gene pairs), and *W. australiana* (27 gene pairs) (Table S2). The *K*_*a*_/*K*_*s*_ ratios of the paralogs and ortholog gene pairs were analyzed to estimate the evolutionary pressure on *SpGARPs*. The *K*_*a*_/*K*_*s*_ ratio ranged from 0.15 to 0.51 (median 0.32) among the *SpGARP* paralogs, suggesting that these genes are under a purifying selection pressure. Similarly, the *K*_*a*_/*K*_*s*_ ratios of *SpGARP* orthologs in *A. thaliana*, *C. esculenta*, *O. sativa* and *W. australiana* ranged from 0.07 to 0.61 (median 0.21), 0.01 to 0.38 (median 0.23), 0.06 to 0.55 (median 0.29), and 0.08 to 0.40 (median 0.19)*,* respectively, indicating that all of them are under the influence of strong purifying or negative selection pressure (Fig. 3A and Table S2). Gene duplication time based on *K*_*s*_ substitution rate in the case of *SpGARP* paralogs was observed in the range of 22.3–88.4 million years ago (MYA), with a median age of 39.7 MYA. The divergence time estimated for the *SpGARP* orthologs in *A. thaliana*, *C. esculenta*, *O. sativa* and *W. australiana* ranged from 57.47 to 119.08 (median 80.75), 22.67 to 75.74 (median 36.54), 30.49 to 166.16 (median 60.78), and 22.68 to 67.65 (median 33.70), respectively (Fig. 3B and Table S2). The syntenic relationship of *SpGARP* orthologs in *C. esculenta* (Fig. 3C) and *O. sativa* (Fig. 3D) showed that group IId (green) and group III (brown) share the majority of orthologs gene pairs.

### Conserved motifs, gene structure and phylogenetic analysis of SpGARPs in *S. polyrhiza*

**Fig. 4:**
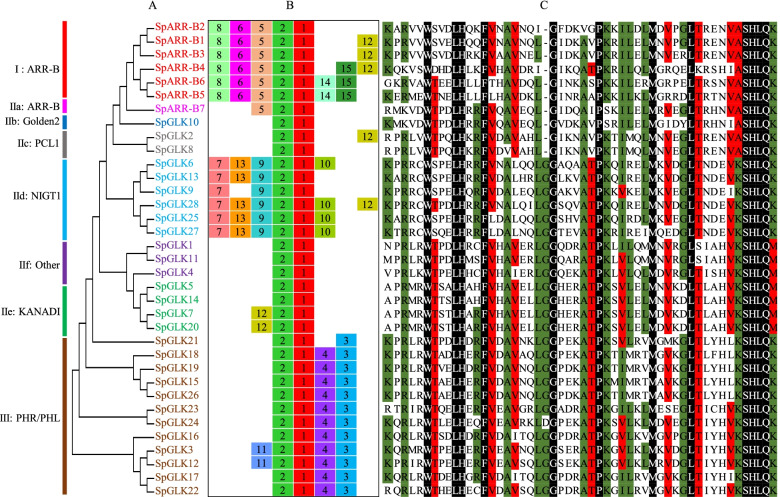
Phylogenetic analysis, conserved motifs (1–15) and the alignment of B-motif of SpGARP proteins. **A** a neighbor-joining (NJ) phylogenetic tree based on the full-length protein sequences. **B** the motifs were ordered manually based on the results of the MEME analysis. **C** Multiple sequence alignment of the domain of B-motif in SpGARPs. PCL1: PHYTOCLOCK1

An unrooted phylogenetic tree was constructed to visualize the evolutionary relationships between GARP members, using 35 SpGARP protein sequences (Fig. 4A). As shown in Fig. 4B, 15 conserved motifs were identified using MEME, motif 1 and 2 were distributed in all the SpGARP proteins, motif 5 in the cluster I and IIa, motif 6/8/14/15 only present in the cluster I, motif 7/9/10/13 present in the cluster of IId, motif 3/4/11 present in group III. The amino acid sequences of B-motif in SpGARP proteins were obtained and aligned ClustalW using displayed in GeneDoc [45]. The B-motif only had one tryptophan (W) residue while the MYB domain had three and MYB-like domain had two W residues [2]. As shown in Fig. 4C, there was only one W residue in the B-motif and a consensus sequence ((A/K)SHLQ(K/M)) in the third helix in SpGARP TFs, as well as the B-motifs in Arabidopsis, rice and tobacco [2, 41, 42].

**Fig. 5:**
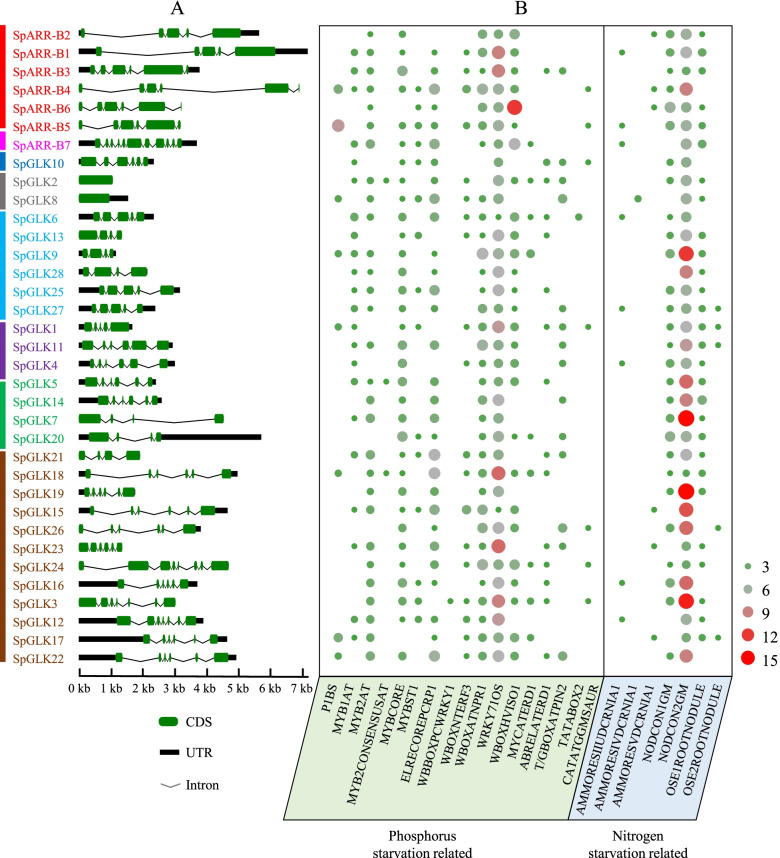
Gene structures and *cis*-elements distribution of *SpGARP* genes. **A** the intron–exon structures of *SpGARP* genes. **B** Frequency of phosphorus and nitrogen starvation related *cis*-elements in the 2-kb pro moter region of *SpGARP* genes

The structure of exons/introns were determined by aligning the genomic DNA sequences and the full-length cDNA of *SpGARP* genes (Fig. 5A). There were 1 (*SpGLK2/8*) to 11 (*SpARR-B7*) exons in *SpGARP* genes, and most of the *SpGARP* genes contained 4–8 exons. The members belonging to the same clade always share the similar gene structure and conserved motifs, such as the members of clade I have 5–6 exons, *SpGLK2* and *SpGLK8* (group IIc) only have one exon, and the *SpNIGT1s* contain 4–5 exons.

### *cis*‑regulatory element analysis of the *SpGARP* gene family

GARP TFs perform diverse functions in plants, such as nutrient sensing, chloroplast development and circadian clock oscillation. The *cis*‑regulatory elements of the promoter regions play important roles in the expression of *GARP* genes for the environmental signals, such as nutrient stress and abiotic stress [40–42]. As shown in Table S3, 246 potential *cis*-acting elements were identified in the promoter regions of *SpGARP* genes. These *cis*-elements were involved in stress and hormone responses. As shown in Fig. 5B, most *SpGARP* genes contained two or more types of P/N starvation related *cis*-elements in their promoter regions. WRKY71OS was the most abundant P starvation related *cis*-elements of *SpGARPs*, as well as NODCON2GM in N starvation related *cis*-elements.

**Fig. 6:**
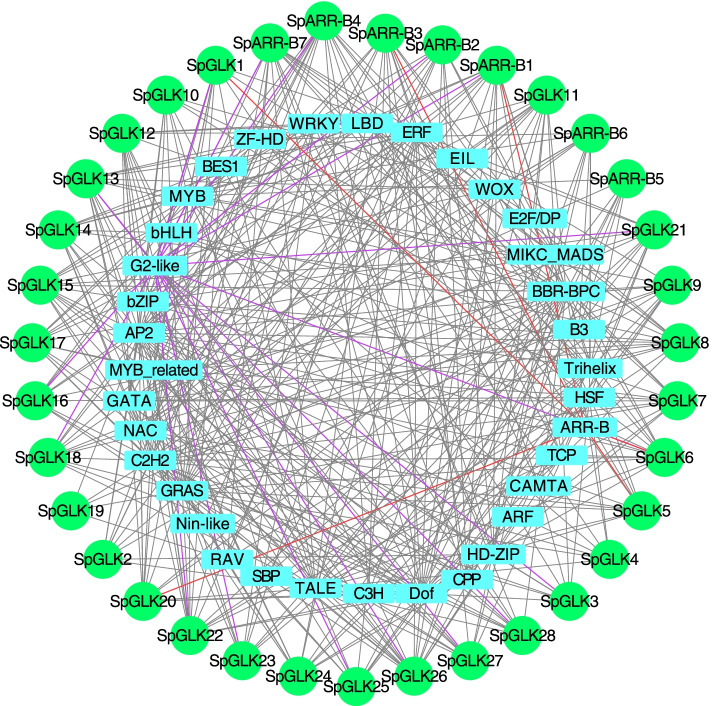
Regulation networks between *SpGARP* and potential transcription factors

We also predicted the TFs that may be involved in regulating the expression of *SpGARP* genes. As shown in Fig. 6, most *SpGARP* genes interacted with multiple TFs, suggesting that they may be involved in many physiological processes. Among these predicted TFs, Apetala2 (AP2), Barley B Recombinant/Basic Pentacysteine (BBR-BPC), C2H2 zinc finger (C2H2), DNA binding with one finger (Dof), Ethylene Response Factor (ERF), Gibberellic-acid Insensitive (GAI)/Repressor of Gai (RGA)/Scarecrow (SCR) (GRAS), G2-like, Three Amino-acid Loop Extension (TALE), and MIKC-type MADS-box (MIKC_MADS) were the most abundant (Table S3 and Fig. 6). Several *SpGARP* genes were regulated by GARP superfamily TFs, six members (*SpARR-B1/3* and *SpGLK1/5/6/20*) contained the ARR-B TF binding site in their promoter regions, and 17 members (3 *SpARR-B* genes and 14 *SpGLK* genes) were regulated by G2-like TFs.

### Transcriptional patterns of *GARP* genes in *S. polyrhiza*

**Fig. 7:**
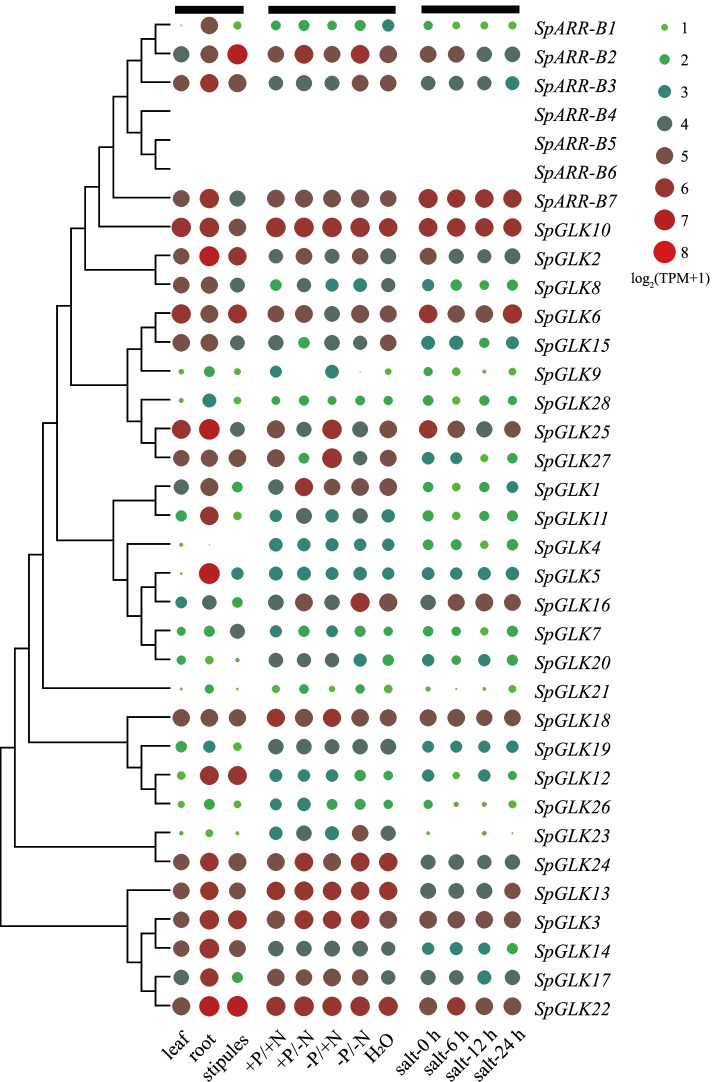
Expression profiles of *SpGARP* genes. The expression data was obtained from RNA-seq data and was expressed as Transcripts Per Kilobase of exon model per Million mapped reads (TPM). The expression data was shown as log_2_ values based on TPM values

The RNA-seq data of *S. polyrhiza* was analyzed to explore the expression patterns of *SpGARP* genes. As shown in Fig. 7, the expression patterns of three members of group I (*SpARR-B4/5/*6) have not been detected in all the transcriptome data. *SpARR-B7*, *SpGLK10* and several members of group III (*SpGLK3/13/18/24/22*) were robust expressed in both fronds and roots as well as under the various conditions. The members of group I (*SpSRR-B1*/2/*3*), IIc (*SpGLK2/8*), IId (*SpGLK9/25/28*), IIe (*SpGLK1/11*), IIf (*SpGLK5/7/16/20*) and III (*SpGLK12/21/26/23*) were expressed differentially between fronds and roots, and most of them were upregulated in roots. NIGT1s were identified as the critical regulators in the plant nutrient sensing, especially in N and P starvation response. All the members of IId subgroup were regulated under N or/and P deprivation. Four out of six members (*SpGLK9/13/25/27*) of group IId were upregulated in the condition of P deprivation (− P/ + N), while downregulated in the condition of N deprivation (+ P/ − N, − P/ − N and H_2_O). PHRs/PHLs were a class of central regulators of PSR, only one member (*SpGLK23*) of group III showed the regulatory expression under nutrient stresses. *SpARR-B2*, *SpGLK2*, and *SpGLK25* were responded to salt stress.

**Fig. 8:**
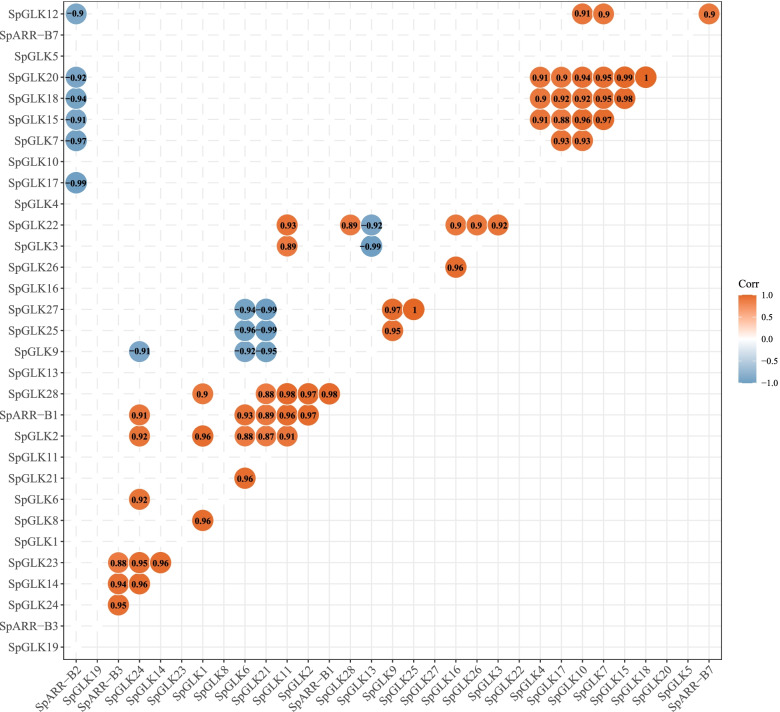
The correlations between the expression patterns of *SpGARP* genes. The significant positive and negative correlation gene pairs (absolute of the correlation value > 0.85, and *p*-value < 0.05) are represented by blue and orange

To further understand the function of *SpGARP* genes under different N and P conditions. The correlations between the expression patterns of *SpGARP* genes were analyzed (Fig. 8). A total of 69 *SpGARP* gene pairs showed correlation expression patterns, including 54 positive correlation and 15 negative correlation *SpGARP* gene pairs. The expressions of three IId members (*SpGLK9*, *SpGLK25*, and *SpGLK27*) were positive correlated, and both negative correlated with *SpGLK6* and *SpGLK21*. Some *SpGARP* genes belonging to different groups presented correlated expression patterns, such as *SpGLK7/10/14/17/18/20*, *SpARR-B1* and *SpGLK2/6/11/21/28*, *SpARR-B3* and *SpGLK14/23/24*.

### The gene regulation network of *SpGARPs* and N/P response genes

**Fig. 9:**
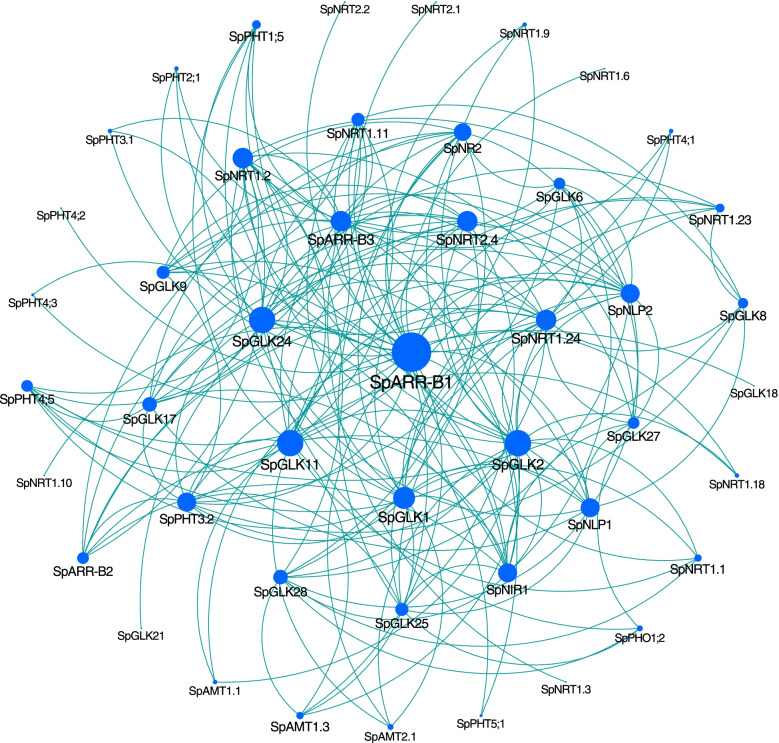
Co-expression network of *SpGARP* and N/P response genes in the turquoise module. 46 differentially expressed *GARP* and N/P response genes with the highest weight are shown in the network, blue circles represent DEGs

To identify the potential cooperative genes of *SpGARPs*, WGCNA was performed using 15 RNA-seq data (PRJNA724886) of *S. polyrhiza* under different N and P conditions., A total of 14,424 genes were used for network construction after removal the low-expression genes (averaged TPM < 1). Those genes were clustered into 18 modules (labeled with different colors), 32 *SpGARP* genes were assigned to 9 modules: 17 genes (*SpARR-B1-3* and *SpGLK1/2/6/8/9/11/14/17/18/21/24/25/27/28*) were assigned to turquoise module, 5 members (*SpGLK7/10/12/20/23*) were assigned to brown module, *SpGLK16* and *SpARR-B7* were assigned to blue module, *SpGLK15* and *SpGLK22* were assigned to red module, and other six modules contained one *SpGARP* member (*SpGLK26*-cyan, *SpGLK4*-green, *SpGLK5*-magenta, *SpGLK13*-salmon, *SpGLK19*-tan, and *SpGLK3*-yellow). The gene co-expression network of *SpGARPs* and N/P response genes in the turquoise module was constructed (Fig. 9). There were 46 genes in the network, including 5 members of NIGT1 subfamily (SpGLK6/9/25/27/28), 10 *PHT* genes, 3 *ammonium transporter* (*AMT*) genes, 11 *NRT1* genes, 3 *NRT2* genes, *NLP1/2*, *nitrate reductase 2* (*NR2*), and *nitrite reductase 1* (*NIR1*).

### Expression analysis of *PHR* and *NIGT1* genes in response to P or/and N deprivation by qRT-PCR

**Fig. 10:**
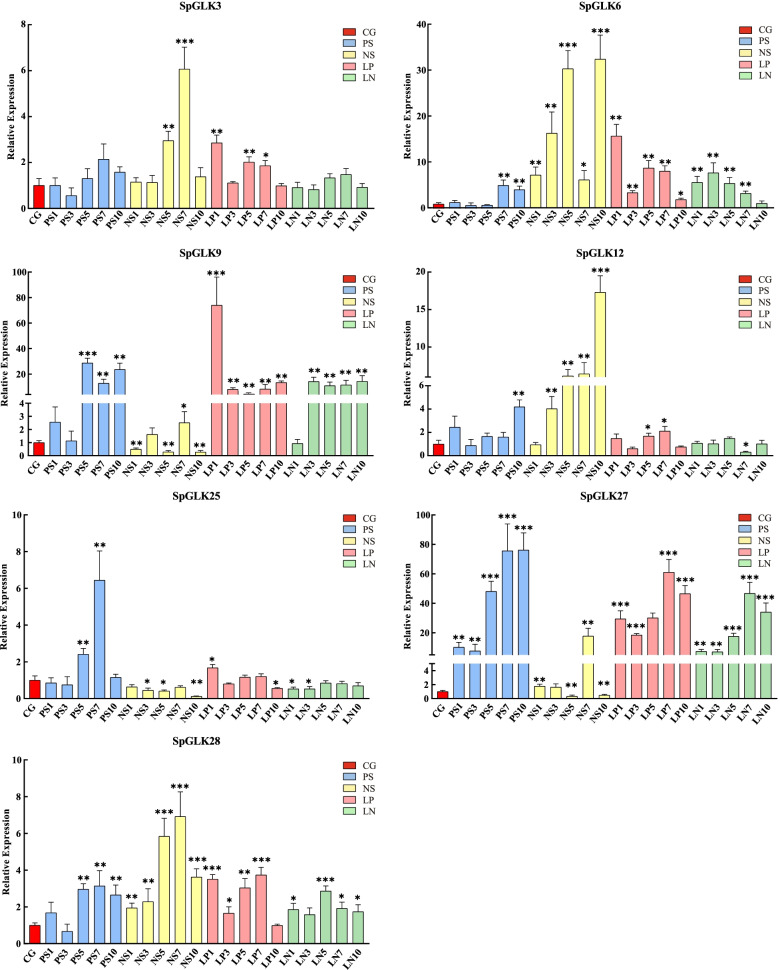
Relative expression of two *PHR* genes and five *NIGT1* genes under nutrient stress conditions. Y axis indicates the relative expression level and X axis represents the samples from different timepoints (0 (Control group, CG), 1, 3, 5, 7 and 10 day) under four treatments (PS, NS, LP and LN). The samples of CG, PS, NS, LP, and LN are represented by red, blue, yellow, pink, and green. Each data point represents mean value ± standard deviation (SD) (*n* = 3). Error bars indicate standard deviation. Asterisks indicate the significant degree of expression level compared to the value of the control (* < 0.05, ** < 0.01, *** < 0.001)

To further clarify the potential abilities of *GARP* genes responding to P and N stresses, the expression profiles of six *NIGT1* subfamily genes (*SpGLK6*, *SpGLK9*, *SpGLK13*, *SpGLK25*, *SpGLK27*, *SpGLK28*) and two *PHR* genes (*SpGLK3*, *SpGLK12*) were verified using qRT-PCR. *SpGLK13* has not been detected in the cDNA samples from both CG and nutrient stress treatments. As showed in Fig. 10, *SpGLK3* was upregulated under N starvation (NS) treatment (NS5 and NS7) and low P (LP) treatment (LP1, LP5 and LP7). The expression of *SpGLK12* was strongly induced by NS treatment. *SpGLK9/25/27* showed the similar expression profiles under PS and NS treatments. Both of them were upregulated under PS and downregulated under NS treatment, with the exception of *SpGLK27* in the NS7. *SpGLK9 and SpGLK27* were also upregulated under LP and LN treatments. The expressions of *SpGLK6/28* were induced under PS, NS, LP and LN treatments, especially under NS treatment.

## Discussion

GARP is a plant specific TF superfamily with diverse functions which have been identified in several species, including Arabidopsis, rice, maize, tobacco, cotton. However, it had not been reported in *S. polyrhiza*, which limited our understanding of the molecular mechanism of this plant to N/P responses. In curent research, we identified 35 SpGARPs belonging to three groups which were further classified into eight subgroups. The numbers of *SpGARP* (35) and *WaGARP* genes (28) were less than those of *A. thaliana* (56), *C. esculenta* (46), *O. sativa* (56), which was consistent with previous report of WRKY TF family in aquatic plants. The reason may be related to the minimal gene set of duckweed genomes (18,708 genes in *S. polyrhiza,* and about 15,000 genes in *W. australiana*) [25, 54, 55]. However, *S. polyrhiza* had more IId members than *C. esculenta*, *O. sativa and W. australiana*, suggesting that NIGT1 subfamily underwent expansion in the adapting to various aquatic environments. It should also be responsible for the high capability of nutrient uptake in *S. polyrhiza*. The SpGARPs that gathered in the same subfamily had the similar gene and protein structures, suggesting the strong evolutionary conservation of *SpGARP* genes [56]. ARR-B family was classified into group I and IIa. However, IIa and IIb showed closer phylogenetic relationship and the similar gene and protein structures, indicating the diverse origins of ARR-B proteins.

GARP members played important roles in many physiological processes as transcriptional regulators [2]. The earlier research found that GARP TFs are involved in chloroplast development [2]. In recent years, their function on nutrient sensing and uptake had gained more attention in plant science [39, 57]. PHRs were the center regulator in the response of Pi starvation, however, the transcriptional level expression of *AtPHR1*, *OsPHR2* had not been improved under the Pi starvation condition [13, 34, 52]. SPX protein withhold PHRs in the cytoplasm under Pi sufficient condition to avoid the toxicity of high Pi, and release PHR proteins into nucleus so as to activate PSR under Pi deficient condition [58, 59]. NIGT1 could regulate the expression of *SPX* genes at transcriptional level, and NIGT1–SPX–PHR cascade mediated the regulation of Pi uptake and starvation signal [38, 46]. In the other hand, NIGT1 TFS acted as transcriptional repressors of N starvation response-related genes [47].

TFs regulated the expression of target genes at the transcriptional level through binding to the *cis*-element in the promoter region. Many types of *cis*-acting elements were present in the promoters of *SpGARP* genes, including abundant of P and N starvation related *cis*-elements, indicating that most *SpGARPs* are involved in hormone and stress responses, especially P and N starvation. The expression patterns of *SpGARP* genes were analyzed in this study, 18 out of 35 *SpGARP* genes were differential expressed between fronds and roots. Furthermore, most members of group IId and III were upregulated in roots. These results indicated that the root might play important roles in mineral sensing and uptake in *S. polyrhiza*. The N or/and P starvation altered the expressions of group I and IId genes, especially *SpGARP6/13/9/25/27*, which was consistent with previous researches in *Arabidopsis* [36, 37]. Salinity stress is one of the major abiotic stresses limiting plant growth and productivity, it also suppresses the growth of *S. polyrhiza* [60]. There are many reports of the GARP genes that responded to salt stress, such as *AtGLK2/5/8/20/23/26/34/43/46* in *A. thaliana* [11], *ZeGLK3* in *Z. mays* [43], 27 *SiGLK* genes in *S. lycopersicum* [61], and *GhGLK55/120* in *G. hirsutum* [40]. In our study, we found that the expression of *SpGLK25* was downregulated under salt stress, which helps to explain why the salt stress affects N/P uptake, and growth rate in plants [60]. The co-expression network showed the regulatory relationship between *SpGARP* and N, P response genes involving N and P sensing, uptake, and assimilation. From the results of qRT-PCR, most of the *SpPHR* and *SpNIGT1* genes were differentially expressed under PS, NS, LP, and LN treatment. Interestingly, *NIGT1s* showed different expression patterns, for example, *SpGLK6/28* were strong induced by NS while *SpGLK9/25/27* were downregulated under NS. The different expression patterns between PS and LP, NS and LN suggested that these genes are involved in N/P sensing.

N and P are crucial for plant growth and food production. Deficiency of N and P mineral nutrients is a huge problem for agriculture. Lots of botanists and agronomists focus their study and research on the adaptation mechanism of plants under the restriction of N or P nutrition. Nazir et al. (2016) found 25 N-deficiency induced proteins between the low-N tolerant and low-N sensitive maize genotypes [62]. Kunar et al. (2018) identified 37 N and P nutrition candidate genes in wheat, including 24 N-use efficiency (NUE) and 13 genes [63]. A total of 12 P-use efficiency (PUE) traits and 136 single nucleotide polymorphisms (SNPs) were identified among 144 diverse mungbean (*Vigna radiata.*) genotypes [64]. Meena et al. (2021) found that the relative expression of some P stress induced (PSI) genes in mungbean accession IC333090 (P-deficiency and drought stress tolerant accession) were significantly higher than that of sensitive accession IC488526, such as *SPX1*, *sulfolipid sulfoquinovosyl diacylglycerol 1* (*SQD1*), *Phosphate1* (PHO1), and *purple acid phosphatase 1* (*PAP1*) [65]. Furthermore, *PHT1*, *PHO1* and *SPX* genes were found to be response to P or/and N deficiency in the aquatic crop *S. polyrhiza* [66, 67].

*S. polyrhiza* is distributed throughout the world, and in the spotlight of plant science, environment remediation, biomass energy, and food security [17]. The discharge of agricultural, industrial and domestic wastewater led to the accumulation of N and P in water, resulting in dramatic water eutrophication and algae blooms. It also destroys the ecological balance of water bodies, lowers the content of dissolved oxygen, deteriorates water quality, and even causes the death of aquatic creatures. Phytoremediation has been recommended as an alternative solution to treat eutrophic wastewater due to its cost-effective, environment friendly and sustainable characteristics. *S. polyrhiza* is widely used in phytoremediation for several outstanding characteristics: fast growth rate, adaptability to a wide range of environmental conditions, high nutrient uptake capacity, and higher proportion of carbohydrate [68]. *S. polyrhiza* can also adapt itself to various nutrient environments by balancing N and P uptake and assimilation. Our comprehensive analysis of *GARP* gene superfamily in *S. polyrhiza* showed that the members of *SpNIGT1* subfamily would be the hub regulators of N and P sensing and acquisition.

## Conclusion

In this study, we identified 35 *GARP* genes in *S. polyrhiza* genome, including 7 *ARR-B* subfamily genes and 28 *GLK* subfamily genes. The gene structures and phylogenetic analysis suggest a complex evolution history of this gene family in *S. polyrhiza*. As the central regulator in P and N nutrients, the *PHR*/*PHL* subfamily genes were transcriptional induced by NS and LN treatments, while the *NIGT1* subfamily genes could respond to P and N stresses, especially *SpGLK9/25/27* which were upregulated under PS treatment and downregulated under NS treatment. This study provides insight into the evolution and function of *GARP* superfamily in *S. polyrhiza*, and facilitates the further functional verification of *SpGARP* genes.

## Materials and methods

### Genome‑wide identification of GARP proteins in *S. polyrhiza*

The genome data *S. polyrhiza* 7498 (55,878) was downloaded from the Comparative Genomics Platform (https://genomevolution.org/CoGe) [25]. The genome of *C. esculenta* (CNP0001082) was downloaded from China National GeneBank DataBase (CNGBdb, https://db.cngb.org/), and the genome of *W. australiana* 8730 was downloaded from Wolffia DB (https://duckweeds.plantprofile.net/) [54]. The published *GARP* superfamily genes (G2-like and ARR-B) of *A. thaliana* [2], and *O. sativa* [41] were downloaded from The Arabidopsis Information Resource (http://www.arabidopsis.org/), and the Rice Genome Annotation Project (http://rice.plantbiology.msu.edu/) respectively [69]. The peptide sequences of GARP from Arabidopsis, and rice were used as queries for protein basic local alignment search tool (BLASTp) analysis against whole-genome sequences in the *S. polyrhiza* 7498 v3 genomes with an e-value cutoff set as 1e^−10^. The HMM profile of B-motif was built using HMMER 3.3.2 based on the identified SpGARPs and used as query for HMMER search (e-value < 1e^−5^). All putative GARP were further checked using Pfam database (phttp://pfam.xfam.org/), Conserved Domains Database (CDD, https://www.ncbi.nlm.nih.gov/cdd/), and Simple Modular Architecture Research Tool database (SMART, http://smart.embl.de/smart/batch.pl). The obtained sequences were aligned with PacBio isoform sequencing data (SRX5321175) of *S. polyrhiza* 7498 to identify the full-length proteins. The *GARP* genes of *C. esculenta* and *W. australiana* were identified using the same methods.

### Basic physicochemical properties and phylogenetic analysis of SpGARP

The molecular weight, isoelectric point and grand average of hydropathicity of SpGARP proteins were calculated for each gene using ExPASy (http://www.expasy.org/tools/) [70]. The subcellular localization of proteins was determined by analysis of the WoLF PSORT (https://wolfpsort.hgc.jp/), CELLO (http://cello.life.nctu.edu.tw/), and Bologna Unified Subcellular Component Annotator (BUSCA, http://busca.biocomp.unibo.it/), and decided based on consensus localization for two or more algorithms [71–73].

The protein sequences of *GARP* TFs from *A. thaliana, C. esculenta, O. sativa*, *S. polyrhiza* and *W. australiana* were aligned using ClustalW [45]. A NJ phylogenetic tree was constructed in MEGAX (http://www.megasoftware.net/) based the multiple sequence alignment with 1000 bootstrap replicates, and displayed using Interactive Tree of Life (iTOL, https://itol.embl.de/) [74, 75].

### Gene duplication and *K*_*a*_/*K*_*s*_ analysis

The information regarding chromosome length and gene locations of *GARP* family genes in *S. polyrhiza* was extracted from the Generic Feature Format (GFF) files. The duplication events were defined based on the collinearity analysis of candidate gene pairs using MCScanX (http://chibba.pgml.uga.edu/mcscan2/) [53]. The synteny analysis of *SpGARP* genes with *CeGARPs* and *OsGARPs* was performed using MCScanX and visualized using Circos (http://mkweb.bcgsc.ca/circos/tableviewer/) [76]. The non-synonymous substitution rate (*K*_*a*_) and synonymous substitution rate (*K*_*s*_) of the duplication and orthologous gene pairs were calculated using PAMLX (http://abacus.gene.ucl.ac.uk/software/paml.html) [77].

### Gene structure analysis and identification of conserved motifs

Multiple Expectation Maximizations for Motif Elicitation (MEME, http://meme-suite.org/) was employed for the analysis of conserved motifs in GARP proteins with the following parameters: maximum number of motifs, 15; motif length, 6 to 50 amino acids [78]. The structure of *GARP* genes, including intron and exon information, was visualized using the online tools Gene Structure Display Server 2.0 (http://gsds.gao-lab.org/index.php/) [79].

### Promoter analysis of *SpGARP* genes

*Cis*-acting elements in the promoter regions of *GARP* genes (2000 bp upstream of the start codon) were predicted and analyzed in New PLACE (https://www.dna.affrc.go.jp/PLACE/?action=newplace/) [80]. The subset of data representing P and N starvation related to *cis* elements was visualized using TBtools [81]. To discover the TFs involving in the regulatory expression of *SpGARP* genes, the online tool PlantRegMAP (http://plantregmap.gao-lab.org/binding_site_prediction.php) was used to predict the potential binding sites of TFs in the promoter regions of *SpGARP* genes [82]. Then, the regulatory networks between *SpGARP* genes and potential TFs were presented using Cytoscape 3.7.0 [83].

### RNA‑seq atlas analysis

The temporal and spatial expression profiles of *SpGARPs* in different tissues/organs (leaves, roots, and stipule, Bio-Project PRJNA557001), under various nutrient stresses (supply with P and N (+ P/ + N), N deprivation (+ P/ − N), P deprivation (− P/ + N), P and N deprivation (− P/ − N), and none nutrient supply (H_2_O) for seven days, PRJNA724886), under salt stress (salt-0 h, salt-6 h, salt-12 h, ssalt-24 h, PRJNA563960) were obtained using the publicly available transcriptome data from NCBI [25, 60]. The heat map showing the correlations between the expression patterns of *SpGARP* genes were generated with the ggplot package in R (version 4.1.2, https://www.R-project.org/). The significant correlated gene pairs should satisfy the following criteria: the absolute of the correlation value > 0.85, *p*-value < 0.05. The genes whose coverage TPM > 1 were filtered and used for WGCNA. Co-expression network modules were identified with the WGCNA package 1.63 in R to generate the signed weighted correlation network, and the network of genes was visualized in Cytoscape 3.7.0 [83, 84].

### Plant materials and treatments

*S. polyrhiza* strain 7498, which was gifted from Duckweed Stock Cooperative (http://www.ruduckweed.org/database.html) and stored in National Aquatic Biological Resource Center (http://www.nabrc.ihb.ac.cn/), was used as the source of plant materials in the study. *S. polyrhiza* was cultivated in liquid half-strength MS solution at pH 5.8, under the conditions of 16 h/8 h photoperiod (day/night), irradiance of 85 µmol photons•m^−2^•s^−1^, and temperature of 25 °C. Ten days later, duckweed was treated in half-strength MS solution without P (PS treatment), half-strength MS solution without N (NS treatment), half-strength MS solution with 1 μM KH_2_PO_4_ (LP treatment), half-strength MS solution with 1 μM NH_4_NO_3_ (LN treatment). The samples were harvested at varied time points (0 (Control group, CG), 1, 3, 5, 7 and 10 days) after the treatment, and immediately frozen in liquid nitrogen and stored at − 80 °C for further analyses. Three samples were collected for each treatment at each time point.

### RNA isolation and qRT‑PCR analysis

The total RNA of duckweed was extracted using Ominiplant RNA Kit (CoWin Biosciences, Beijing, China). From total RNA, first-strand cDNA was synthesized using a PrimeScript™ RT reagent Kit (TaKaRa, Dalian, China). The primers of *SpGARP* genes were presented in Table S4. qRT-PCR program was performed using the same methods in previous study [85]. Each reaction was analyzed in triplicate and the 2^−△△CT^ method was used to analyze the data [86]. The qRT–PCR results were statistically analyzed using SPSS 25.0 software. Significance differences were determined by one-way ANOVA and a Fisher’s LSD test at the *p* < 0.05.

## Supplementary Information

**Additional file 1:** **Table S1.** List of GARP superfamily members identified genome-wide in Colocasia esculenta and Wolffia Australiana. **Table S2.** The Ka/Ks and divergence time of SpGARP paralogs and orthologs gene pairs. **Table S3.** The frequency of 245 cis-regulatory elements in the 2000 bp promoter region of GARP genes in giant duckweed, scanned in New PLACE database. **Table S4.** Primer sequences used in qRT-PCR.

## Data Availability

RNA-seq data of *S. polyrhiza* under nutrient (N and P) deficiency have been deposited in NCBI SRA with the accession number PRJNA724886. RNA-seq data of *S. polyrhiza* tissues/organs are available in NCBI under accession number PRJNA557001. RNA-seq data of *S. polyrhiza* under salt stress are available in NCBI under accession number PRJNA563960. The expression data of *SpPHR* and *SpNIGT1* genes generated by qRT-PCR are available from the corresponding authors when needed. All other data generated or analyzed during this study are included in this article and its supplementary information files.

## Abbreviations

ARR-B: Type-B authentic response regulators
GARP: *GOLDEN2-ARR-B-Psr1*
GRAVY: Grand average of hydropathicity
GLK: *GOLDEN2-like*
HRS1: *Hypersensitivity to Low Phosphate-Elicited Primary Root Shortening 1*
HHO: *HRS1 Homolog*
HMM: Hidden Markov Model
K_a_/K_s_: The ratio of the number of nonsynonymous substitutions per nonsynonymous site (*K*_*a*_) to the number of synonymous substitutions per synonymous site (*K*_*s*_)
MW: Protein molecular weight
NIGT1: *Nitrate-Inducible Garp-Type Transcriptional Repressor 1*
PCL1: PHYTOCLOCK1
PHR1: *Phosphate Starvation Response 1*
pI: Isoelectric point
Pi: Inorganic phosphate
qRT-PCR: Quantitative Real-time PCR
TPM: Transcripts Per Million
WGCNA: Weighted correlation network analysis
