# Supplementary material for: Identification and expression analysis of GARP superfamily genes in response to nitrogen and phosphorus stress in Spirodela polyrhiza
Source: BMC Plant Biol. 2022 Jun 25;22:308. doi: 10.1186/s12870-022-03696-5 (PMC9233324; doi:10.1186/s12870-022-03696-5)
Supplement: Supplementary file 1 — Additional file 1: Table S1. List of GARP superfamily members identified genome-wide in Colocasia esculenta and Wolffia Australiana. Table S2. The Ka/Ks and divergence time of SpGARP paralogs and orthologs gene pairs. Table S3. The frequency of 245 cis-regulatory elements in the 2000 bp promoter region of GARP genes in giant duckweed, scanned in New PLACE database. Table S4. Primer sequences used in qRT-PCR. [file 12870_2022_3696_MOESM1_ESM.zip › Table S4.docx]

**Table S4. Primer sequences used in qRT-PCR.**

| Gene | Forward primer (5’-3’) | Reverse primer (5’-3’) |
| --- | --- | --- |
| SpGLK3 | TGATTCTGATGCAACTGCCACTGAG | TGCTTGGCTGGTGCGTTACTTG |
| SpGLK6 | ATGCTCATCCTCAACGACACGAT | TGACCTTGTCCCTGAAATCCTTCTC |
| SpGLK9 | GCTCCCTCTATGCGTCATCATCC | GCGGTGGTGGTGGACGAATT |
| SpGLK12 | ATCATCACCACCGCCATCTTCAG | ACATTCATCAGCTCCAGTACCGATT |
| SpGLK13 | CTAACAGCGGCGACAGCAAGA | GCGGCGGCAAGAAAGAGAGA |
| SpGLK25 | GCCACCGCCAAGAGCAGATTA | GCCGAGAAGTCTCATCATCCTCAT |
| SpGLK27 | AGTCCACCGCCACATTACTCACT | TCCCTCTTGCTCCTCTGGTCTCA |
| SpGLK28 | ATCGCCGACCAGTCTGACCA | CAGTTGCTGTCTGTAGGCTTCCA |
| SpACT1 | CCAGATCATGTTCGAGACCTTCAAC | GGAGGGCGTATCCTTCGTAGATG |
