# Supplementary material for: Identification and expression analysis of GARP superfamily genes in response to nitrogen and phosphorus stress in Spirodela polyrhiza
Source: BMC Plant Biol. 2022 Jun 25;22:308. doi: 10.1186/s12870-022-03696-5 (PMC9233324; doi:10.1186/s12870-022-03696-5)
Supplement: Supplementary file 1 — Additional file 1: Table S1. List of GARP superfamily members identified genome-wide in Colocasia esculenta and Wolffia Australiana. Table S2. The Ka/Ks and divergence time of SpGARP paralogs and orthologs gene pairs. Table S3. The frequency of 245 cis-regulatory elements in the 2000 bp promoter region of GARP genes in giant duckweed, scanned in New PLACE database. Table S4. Primer sequences used in qRT-PCR. [file 12870_2022_3696_MOESM1_ESM.zip › Table S2.docx]

**Table S2. The *K_a_*/*K_s_* and divergence time of *SpGARP* paralogs and orthologs gene pairs.**

| Seq_1 |  | Seq_2 | *K_a_* | K_s_ | *K_a_/K_s_* | Estimated gene duplication time (MYA) |
| --- | --- | --- | --- | --- | --- | --- |
| SpGLK5 |  | SpGLK14 | 0.47273 | 1.450742 | 0.325854 | 48.35806 |
| SpGLK17 |  | SpGLK22 | 0.234605 | 0.975943 | 0.240388 | 32.53143 |
| SpGLK10 |  | SpGLK15 | 1.069659 | 3.621235 | 0.295385 | 120.7078 |
| SpGLK15 |  | SpGLK26 | 0.299826 | 1.298611 | 0.230882 | 43.28704 |
| SpGLK25 |  | SpGLK27 | 0.373586 | 1.267987 | 0.294629 | 42.26623 |
| SpGLK3 |  | SpGLK12 | 0.406598 | 2.652376 | 0.153296 | 88.41253 |
| SpGLK9 |  | SpGLK13 | 0.460494 | 1.17618 | 0.391517 | 39.20598 |
| SpGLK6 |  | SpGLK13 | 0.340872 | 0.668537 | 0.509878 | 22.28455 |
| SpGLK6 |  | SpGLK9 | 0.383779 | 1.190017 | 0.322499 | 39.66722 |
| SpARR-B5 |  | SpARR-B6 | 0.396468 | 0.990035 | 0.400459 | 33.00117 |
| SpGLK8 |  | AT5G59570.2 | 0.434551 | 2.338624 | 0.185815 | 77.95413 |
| SpGLK17 |  | AT2G01060.1 | 0.24773 | 2.353335 | 0.105268 | 78.4445 |
| SpGLK24 |  | AT3G13040.1 | 2.192271 | 3.572457 | 0.613659 | 119.0819 |
| SpGLK1 |  | AT2G38300.1 | 0.729309 | 2.09232 | 0.348565 | 69.74399 |
| SpGLK14 |  | AT5G42630.1 | 0.586977 | 2.49156 | 0.235586 | 83.052 |
| SpGLK22 |  | AT2G01060.1 | 0.232616 | 3.216518 | 0.072319 | 107.2173 |
| SpGLK27 |  | AT1G13300.1 | 0.511058 | 1.723401 | 0.29654 | 57.4467 |
| SpGLK3 |  | AT2G20400.2 | 0.582537 | 3.534123 | 0.164832 | 117.8041 |
| SpGLK4 |  | CeGLK15 | 0.205362 | 0.883376 | 0.232474 | 29.44588 |
| SpGLK1 |  | CeGLK35 | 0.24676 | 1.010857 | 0.24411 | 33.69522 |
| SpGLK11 |  | CeGLK28 | 0.335012 | 1.306472 | 0.256425 | 43.54907 |
| SpGLK5 |  | CeGLK8 | 0.353372 | 1.087444 | 0.324956 | 36.24815 |
| SpARR-B3 |  | CeARR-B6 | 0.235181 | 1.849814 | 0.127138 | 61.66046 |
| SpARR-B2 |  | CeARR-B10 | 0.367071 | 1.937207 | 0.189485 | 64.57358 |
| SpARR-B1 |  | CeARR-B4 | 0.307143 | 1.609789 | 0.190797 | 53.65962 |
| SpGLK8 |  | CeGLK24 | 0.259177 | 1.081502 | 0.239645 | 36.05006 |
| SpGLK2 |  | CeGLK31 | 0.211164 | 0.680144 | 0.310469 | 22.67148 |
| SpGLK23 |  | CeGLK1 | 0.339871 | 1.457035 | 0.233262 | 48.56782 |
| SpGLK16 |  | CeGLK5 | 0.191321 | 1.078392 | 0.177413 | 35.94641 |
| SpGLK17 |  | CeGLK23 | 0.22809 | 1.131848 | 0.20152 | 37.72826 |
| SpGLK18 |  | CeGLK25 | 0.151544 | 1.438247 | 0.105367 | 47.94158 |
| SpGLK15 |  | CeGLK16 | 0.252591 | 1.516497 | 0.166562 | 50.5499 |
| SpGLK25 |  | CeGLK20 | 0.230463 | 0.937922 | 0.245716 | 31.26407 |
| SpGLK27 |  | CeGLK7 | 0.299913 | 0.938668 | 0.319509 | 31.28894 |
| SpGLK3 |  | CeGLK34 | 0.208624 | 0.992615 | 0.210177 | 33.08717 |
| SpGLK9 |  | CeGLK3 | 0.35098 | 0.927808 | 0.378289 | 30.92693 |
| SpGLK9 |  | CeGLK14 | 0.378717 | 1.329956 | 0.284759 | 44.33187 |
| SpGLK10 |  | CeGLK36 | 0.111544 | 1.046921 | 0.106545 | 34.89735 |
| SpGLK7 |  | CeGLK19 | 0.520334 | 1.723544 | 0.301897 | 57.45147 |
| SpGLK20 |  | CeGLK26 | 0.293398 | 1.104898 | 0.265543 | 36.82992 |
| SpGLK19 |  | CeGLK13 | 0.126833 | 1.035562 | 0.122478 | 34.51873 |
| SpGLK28 |  | CeGLK11 | 0.265951 | 1.146505 | 0.231966 | 38.21682 |
| SpARR-B7 |  | CeARR-B9 | 0.286662 | 1.348595 | 0.212564 | 44.95315 |
| SpGLK14 |  | CeGLK8 | 0.318982 | 1.121235 | 0.284491 | 37.37451 |
| SpARR-B2 |  | CeARR-B8 | 0.320291 | 1.263522 | 0.25349 | 42.11741 |
| SpARR-B1 |  | CeARR-B1 | 0.307143 | 1.609789 | 0.190797 | 53.65962 |
| SpGLK8 |  | CeGLK33 | 0.154646 | 0.94953 | 0.162866 | 31.651 |
| SpGLK2 |  | CeGLK10 | 0.126633 | 0.811247 | 0.156096 | 27.04157 |
| SpGLK22 |  | CeGLK23 | 0.173687 | 1.315024 | 0.132079 | 43.83414 |
| SpGLK26 |  | CeGLK16 | 0.130484 | 0.851247 | 0.153286 | 28.3749 |
| SpGLK13 |  | CeGLK3 | 0.320744 | 0.854706 | 0.375269 | 28.4902 |
| SpGLK13 |  | CeGLK14 | 0.32002 | 0.893387 | 0.35821 | 29.77956 |
| SpGLK7 |  | CeGLK32 | 0.516496 | 1.674801 | 0.308392 | 55.82671 |
| SpGLK20 |  | CeGLK30 | 0.123078 | 0.984055 | 0.125072 | 32.80182 |
| SpARR-B1 |  | CeARR-B7 | 0.212791 | 1.588929 | 0.133921 | 52.96428 |
| SpGLK8 |  | CeGLK17 | 0.121455 | 0.831113 | 0.146136 | 27.70375 |
| SpGLK6 |  | CeGLK3 | 0.193048 | 0.684609 | 0.281983 | 22.82029 |
| SpGLK6 |  | CeGLK14 | 0.264821 | 0.848291 | 0.312181 | 28.27636 |
| SpGLK7 |  | CeGLK12 | 0.403956 | 1.651777 | 0.244558 | 55.05923 |
| SpARR-B1 |  | CeARR-B2 | 0.188716 | 2.272308 | 0.083051 | 75.7436 |
| SpGLK4 |  | OsGLK51 | 0.674042 | 2.222378 | 0.303297 | 74.07926 |
| SpGLK1 |  | OsGLK45 | 0.694197 | 2.56298 | 0.270855 | 85.43266 |
| SpGLK11 |  | OsGLK48 | 0.55203 | 2.362654 | 0.233648 | 78.75514 |
| SpGLK5 |  | OsGLK18 | 0.652061 | 2.243077 | 0.290699 | 74.76924 |
| SpGLK8 |  | OsGLK5 | 0.343148 | 1.150075 | 0.29837 | 38.33584 |
| SpGLK2 |  | OsGLK21 | 0.387502 | 1.108576 | 0.349549 | 36.95255 |
| SpGLK23 |  | OsGLK20 | 0.506616 | 1.823317 | 0.277854 | 60.77722 |
| SpGLK16 |  | OsGLK23 | 0.323099 | 2.394051 | 0.134959 | 79.8017 |
| SpGLK17 |  | OsGLK40 | 0.282575 | 4.984932 | 0.056686 | 166.1644 |
| SpGLK18 |  | OsGLK35 | 0.301422 | 2.576797 | 0.116975 | 85.89323 |
| SpGLK25 |  | OsGLK10 | 0.546908 | 1.236112 | 0.442442 | 41.20375 |
| SpGLK9 |  | OsGLK46 | 0.47592 | 1.263682 | 0.376614 | 42.12272 |
| SpGLK13 |  | OsGLK46 | 0.511909 | 1.066201 | 0.480124 | 35.54003 |
| SpGLK6 |  | OsGLK46 | 0.500708 | 1.1141 | 0.449429 | 37.13667 |
| SpGLK10 |  | OsGLK2 | 0.337259 | 1.195029 | 0.282218 | 39.8343 |
| SpGLK7 |  | OsGLK37 | 0.736009 | 2.300888 | 0.31988 | 76.69627 |
| SpGLK20 |  | OsGLK34 | 0.471598 | 1.163215 | 0.405426 | 38.77382 |
| SpGLK19 |  | OsGLK29 | 0.309832 | 1.712152 | 0.180961 | 57.07172 |
| SpGLK28 |  | OsGLK52 | 0.361514 | 1.198238 | 0.301705 | 39.94128 |
| SpGLK4 |  | OsGLK43 | 0.636989 | 1.366602 | 0.466111 | 45.5534 |
| SpGLK1 |  | OsGLK44 | 0.590892 | 3.596436 | 0.164299 | 119.8812 |
| SpGLK11 |  | OsGLK25 | 0.456175 | 2.490431 | 0.183171 | 83.01437 |
| SpGLK5 |  | OsGLK18 | 0.652061 | 2.243077 | 0.290699 | 74.76924 |
| SpGLK17 |  | OsGLK36 | 0.298052 | 2.960291 | 0.100683 | 98.67636 |
| SpGLK22 |  | OsGLK36 | 0.282552 | 3.302971 | 0.085545 | 110.099 |
| SpGLK27 |  | OsGLK10 | 0.542043 | 1.358718 | 0.398937 | 45.2906 |
| SpGLK3 |  | OsGLK32 | 0.472005 | 2.019776 | 0.233692 | 67.32587 |
| SpGLK9 |  | OsGLK17 | 0.492024 | 1.093433 | 0.449981 | 36.44775 |
| SpGLK13 |  | OsGLK17 | 0.479336 | 0.934593 | 0.512882 | 31.1531 |
| SpGLK6 |  | OsGLK17 | 0.50345 | 0.914606 | 0.550455 | 30.48686 |
| SpGLK10 |  | OsGLK1 | 0.352432 | 1.791297 | 0.196747 | 59.70989 |
| SpGLK7 |  | OsGLK41 | 0.618183 | 2.001795 | 0.308814 | 66.7265 |
| SpGLK19 |  | OsGLK8 | 0.291516 | 1.71188 | 0.17029 | 57.06266 |
| SpGLK4 |  | OsGLK49 | 0.666285 | 1.889145 | 0.352691 | 62.97149 |
| SpARR-B3 |  | OsRRB5 | 0.488946 | 4.024254 | 0.1215 | 134.1418 |
| SpGLK17 |  | OsGLK28 | 0.291418 | 2.005045 | 0.145342 | 66.83483 |
| SpGLK22 |  | OsGLK28 | 0.335925 | 2.001429 | 0.167843 | 66.71428 |
| SpGLK4 |  | OsGLK19 | 0.481254 | 1.338356 | 0.359586 | 44.61186 |
| SpGLK4 |  | WaGLK1 | 0.296898 | 0.90544 | 0.327904 | 30.18133 |
| SpGLK1 |  | WaGLK10 | 0.343057 | 1.099945 | 0.311885 | 36.66483 |
| SpGLK11 |  | WaGLK2 | 0.292642 | 1.332786 | 0.219572 | 44.4262 |
| SpGLK14 |  | WaGLK12 | 0.209814 | 1.267025 | 0.165595 | 42.23416 |
| SpARR-B1 |  | WaARR-B5 | 0.190241 | 1.212815 | 0.156859 | 40.42715 |
| SpGLK8 |  | WaGLK6 | 0.181728 | 0.937773 | 0.193786 | 31.25911 |
| SpGLK2 |  | WaGLK22 | 0.187465 | 0.680416 | 0.275516 | 22.68052 |
| SpGLK22 |  | WaGLK8 | 0.139653 | 1.371812 | 0.101802 | 45.72706 |
| SpGLK18 |  | WaGLK5 | 0.12552 | 1.518179 | 0.082678 | 50.60596 |
| SpGLK15 |  | WaGLK18 | 0.333346 | 1.772791 | 0.188035 | 59.09303 |
| SpGLK25 |  | WaGLK3 | 0.181472 | 1.223731 | 0.148294 | 40.79104 |
| SpGLK27 |  | WaGLK14 | 0.2033 | 1.267388 | 0.160408 | 42.24626 |
| SpGLK3 |  | WaGLK11 | 0.278382 | 1.345834 | 0.206847 | 44.86114 |
| SpGLK12 |  | WaGLK13 | 0.378305 | 1.247391 | 0.303277 | 41.57971 |
| SpGLK10 |  | WaGLK15 | 0.088845 | 0.895891 | 0.09917 | 29.86304 |
| SpGLK7 |  | WaGLK7 | 0.576822 | 1.703316 | 0.338647 | 56.7772 |
| SpGLK20 |  | WaGLK4 | 0.121063 | 1.065461 | 0.113625 | 35.51537 |
| SpARR-B7 |  | WaARR-B4 | 0.419876 | 1.039904 | 0.403765 | 34.66346 |
| SpGLK5 |  | WaGLK17 | 0.275488 | 1.073579 | 0.256607 | 35.78596 |
| SpGLK14 |  | WaGLK17 | 0.477872 | 2.02958 | 0.235454 | 67.65266 |
| SpARR-B1 |  | WaARR-B1 | 0.190241 | 1.212815 | 0.156859 | 40.42715 |
| SpGLK2 |  | WaGLK21 | 0.187465 | 0.680416 | 0.275516 | 22.68052 |
| SpGLK15 |  | WaGLK18 | 0.333346 | 1.772791 | 0.188035 | 59.09303 |
| SpGLK10 |  | WaGLK23 | 0.088845 | 0.906754 | 0.097982 | 30.22514 |
| SpARR-B7 |  | WaARR-B3 | 0.148021 | 0.86846 | 0.170441 | 28.94868 |
| SpGLK2 |  | WaGLK16 | 0.187465 | 0.680416 | 0.275516 | 22.68052 |
| SpGLK10 |  | WaGLK20 | 0.108898 | 0.926736 | 0.117507 | 30.89121 |
